# Weaning in INfant hip DYsplasia (WINDY) study: weaning of brace treatment versus immediate cessation for developmental dysplasia of the hip: study protocol for a randomized multicentre feasibility study

**DOI:** 10.1302/2633-1462.77.BJO-2026-0074.R1

**Published:** 2026-07-09

**Authors:** Joanna Craven, Richard Kirk, Xavier L. Griffin, Elizabeth Conroy, Eleanor C. Carpenter, Lucy Llewellyn-Stanton, Alexander Aarvold, Daniel C. Perry

**Affiliations:** 1 University of Liverpool, Liverpool, UK; 2 Wales Deanery, Nantgarw, UK; 3 Queen Mary University of London Barts and The London School of Medicine and Dentistry, London, UK; 4 Barts Health NHS Trust, London, UK; 5 University of Oxford NDORMS, Oxford, UK; 6 Noah’s Ark Children’s Hospital for Wales, Cardiff, UK; 7 Oxford University Hospitals NHS Foundation Trust, Oxford, UK; 8 University of Southampton, Southampton, UK; 9 Southampton Children’s Hospital, Southampton, UK; 10 Alder Hey Children’s Hospital, Liverpool, UK

**Keywords:** DDH, Weaning, Protocol, WINDY, Developmental dysplasia of the hip (DDH), brace treatment, clinicians, randomized controlled trials, hips, Pavlik harness, multicentre randomized controlled trial, visual analogue scales, Orthopaedic Surgery, clinical outcomes

## Abstract

**Aims:**

Pavlik harness (brace) treatment is the cornerstone of management for infants with developmental dysplasia of the hip (DDH). However, there is wide variation in how the harness is discontinued, with some clinicians stopping immediately and others using gradual reduction (‘weaning’), reflecting the absence of high-quality comparative evidence. WINDY (Weaning in INfant hip Dysplasia) is a multicentre randomized feasibility trial comparing a standardized weaning regimen with immediate cessation. The primary aim is to determine whether a definitive multicentre randomized controlled trial (RCT) is feasible. Secondary aims are to assess parental engagement, evaluate the acceptability and measurement properties of the Evaluation Measure for BRACe Experience (EMBRACE), and explore whether routinely collected Smart4NIPE data can be used for trial outcomes.

**Methods:**

WINDY is a multicentre, parallel-group feasibility RCT with 1:1 allocation, stratified by site. Infants treated with a Pavlik harness who demonstrate ultrasound normalization (hip centred; α angle ≥ 60°) are eligible. Following consent, infants are randomized to either four weeks of night-time-only harness wear (minimum 10 hours per night) or immediate cessation. Follow-up is 12 weeks and aligned with routine care. Parents complete electronic questionnaires, including EMBRACE, at baseline, two, four, and six weeks post-randomization. Feasibility outcomes include recruitment, proportion randomized, parental engagement, and compliance. EMBRACE measurement properties and the feasibility of using routinely collected national data will also be assessed.

**Discussion:**

This study will determine key feasibility parameters for a definitive multicentre RCT comparing harness weaning with immediate cessation in DDH, including recruitment, intervention acceptability, and parent-reported data completion. It will also provide further evaluation of the EMBRACE measure to support family-centred outcomes and assess whether routinely collected Smart4NIPE data can be used for trial outcome capture. As a feasibility study, analyses will be descriptive and will inform the design and delivery of a future definitive trial.

Cite this article: *Bone Jt Open* 2026;7(7):900–906.

## Introduction

Developmental dysplasia of the hip (DDH) describes a spectrum of abnormal hip development ranging from mild acetabular dysplasia to frank dislocation.^1,2^ Approximately 1% of infants are affected, with around one in 1,000 presenting with a dislocated hip.^3^ Early diagnosis and treatment are associated with improved outcomes and reduced risk of long-term complications, including early osteoarthritis and the need for hip arthroplasty in early adulthood.^4,5^ Pavlik harness (brace) treatment is the cornerstone of management for infants under six months with reducible hips and is widely used in the UK.^6-8^

Despite widespread use, there is variation in how full-time brace wear is discontinued once ultrasound normalization is achieved.^9^ Some clinicians stop the harness immediately, whereas others gradually reduce wear (‘weaning’). Surveys demonstrate that approximately two-thirds of UK clinicians use immediate cessation, while one-third use weaning, although internationally, weaning is more common.^8,10^ Consensus guidance is inconsistent, and there are no randomized controlled trials (RCTs) comparing these approaches.^11^ Existing comparative studies are small and observational, with no clear evidence favouring either strategy.^12,13^

Brace treatment is generally considered low risk but is associated with complications including avascular necrosis, femoral nerve palsy, skin irritation, and caregiver burden. Parents report substantial impacts on family life, including sleep disruption, feeding difficulties, bonding challenges, and emotional stress.^14,15^ Minimizing brace duration without compromising outcomes may therefore benefit both infants and families.

### Rationale

Standardizing treatment pathways in DDH has been identified as a research priority by the British Society of Children’s Orthopaedic Surgery and through a James Lind Alliance priority setting partnership.^16,17^ Proponents of weaning suggest that gradual reduction may reduce relapse and residual acetabular dysplasia, whereas opponents argue that continued brace wear after hip normalization offers no additional benefit and increases burden on families and healthcare services. Clinical equipoise therefore exists.

Before undertaking a definitive RCT, key uncertainties must be addressed. These include whether clinicians and families will accept randomization, whether a standardized weaning intervention is deliverable, and whether recruitment rates are sufficient. In addition, family-centred outcomes have been identified as essential in DDH research, leading to the development of the Evaluation Measure for BRACe Experience (EMBRACE), a new parent-reported outcome measure requiring further evaluation.^14,18^ The feasibility of using routinely collected screening data from the national Newborn Infant Physical Examination (NIPE) programme to support trial data collection also requires assessment, as the use of the database (Smart4NIPE) could reduce research burden and improve trial efficiency.

### Aims and objectives

The primary aim of the WINDY (Weaning in INfant hip Dysplasia) study is to determine the feasibility of conducting a definitive multicentre RCT comparing weaning of brace treatment with immediate cessation in infants with DDH. The objectives are:

1. To assess the acceptability of the trial design, including recruitment rates and proportion of eligible infants randomized.
2. To evaluate parental engagement, including compliance with allocated treatment and completion of parent-reported outcome measures.
3. To assess the reliability, validity, and acceptability of the EMBRACE measure.
4. To determine the accessibility, completeness, and accuracy of routinely collected Smart4NIPE data for research use.
5. To explore clinical outcomes, including re-intervention rates, acetabular development, adverse events (AEs), and healthcare use.

## Methods

WINDY is a multicentre, prospective, parallel-group, pragmatic, randomized feasibility trial with 1:1 allocation comparing brace weaning with immediate cessation in infants with DDH. The study is non-blinded due to the nature of the interventions. The trial aims to recruit 60 participants over an approximate six-month recruitment period (anticipated six to nine months overall, including site set-up). The study is registered with the International Standard RCT Number (ISRCTN) as ISRCTN11590338.

### Study setting

The trial will be conducted in a minimum of three NHS hospitals in the UK. Eligible infants will be identified through routine DDH outpatient clinics and screening pathways.

### Study participants

Infants are eligible if Pavlik harness treatment was initiated under six months of age, full-time harness wear has been undertaken, ultrasound demonstrates hip centring with an α angle equal to or greater than 60°, and if the parent or legal guardian is willing and able to provide consent. Infants are ineligible if they have a known or suspected neuromuscular condition, or if the parent or guardian is unable to adhere to study procedures or complete questionnaires.

### Sample size

As a feasibility study, a formal power calculation has not been undertaken. A target sample size of 60 infants was selected to estimate recruitment rates, intervention acceptability, and data completeness across participating sites, and to inform the design of a future definitive trial.

### Participant recruitment

Eligible infants will be identified by the treating clinical team during routine appointments. Parents will receive written information about the study and have the opportunity to discuss participation with the research team. Informed consent will be obtained from the parent or legal guardian using an approved electronic consent process. Screening logs will record eligibility, recruitment, and reasons for non-participation where available.

### Randomization and blinding

Following consent and baseline data collection, infants will be randomized on a 1:1 basis using a secure web-based randomization system (Research Electronic Data Capture; REDCap), stratified by recruiting site with permuted blocks of varying sizes. Due to the nature of the interventions, parents and clinicians will not be blinded to allocation.

## Interventions

### Weaning intervention

Infants randomized to the intervention will continue Pavlik harness wear at night-time (minimum ten hours per night) for four weeks. This regimen reflects commonly used clinical practice and was informed by clinician surveys.^9^ Parents will receive written instructions outlining the weaning schedule.

### Immediate cessation

Infants randomized to the control group will stop Pavlik harness wear immediately following randomization. All infants will continue routine clinical follow-up according to local practice. No additional imaging or hospital visits are mandated by the study protocol.

### Follow-up and outcome assessment

Parents will complete electronic questionnaires at baseline and at two, four, and six weeks post-randomization, including EMBRACE, visual analogue scales assessing family, parent, and infant impact, and self-reported compliance. Clinical data, including routine ultrasound findings and re-intervention, will be collected up to 12 weeks post-randomization.

Feasibility outcomes include recruitment rate per centre, proportion of eligible infants randomized, parental engagement, compliance with allocation, and completeness of outcome data. EMBRACE will be evaluated for reliability, validity, and acceptability. Subject to approvals, routinely collected Smart4NIPE data will be compared with trial data to assess accessibility, completeness, and accuracy.

## Outcomes

### Primary outcome

The primary outcome is feasibility of the trial design, assessed by recruitment rate per centre per month, and the proportion of eligible infants who are randomized. Screening logs will record numbers approached, reasons for exclusion, and reasons for declining participation where available.

### Secondary outcomes

Feasibility and measurement outcomes: Secondary feasibility outcomes include parental engagement, assessed by questionnaire response rates and self-reported compliance with allocation, completeness of outcome data, and study retention. EMBRACE will be evaluated for reliability (floor and ceiling effects, internal consistency, and test–retest reliability), construct validity (correlation with visual analogue scale measures of family, parent, and infant impact), and acceptability (completion time and missing data).

The feasibility of using routinely collected Smart4NIPE data for trial outcomes will also be assessed, including accessibility, completeness, and accuracy compared with trial database records.

Clinical and exploratory outcomes: Exploratory clinical outcomes include acetabular development based on routine imaging up to 12 weeks post-randomization, re-intervention rates (further bracing or surgery), AEs, and the number of DDH-related hospital attendances. Family impact will be explored using EMBRACE scores over time.

The study objectives and outcome measures are summarized in Table I.

**Table I.** WINDY study objectives and outcome measures.

	Objectives	Outcome measures
Feasibility objectives and outcomes	Acceptance of the study design	Recruitment rate per month per centre
		% eligible infants randomized. Reason for exclusion/ declining to participate
	Parental engagement	Parent-reported compliance and availability of parent-reported data
	EMBRACE reliability	Floor and ceiling effects (%), test-retest and Cronbach’s α
	EMBRACE validity	Correlation between EMBRACE and VAS for impact to family, parent, and infant
	EMBRACE acceptability	Time taken to complete survey and missing data (%)
	Accessibility of Smart4NIPE data	Availability of routinely collected data within the Smart4NIPE system for research purposes
	Accuracy and completeness of Smart4NIPE data	Accuracy and completeness of Smart4NIPE data compared trial data recorded within trial database (REDCap) (%)
Exploratory outcomes and objectives	Objectives	Outcome measures
	Acetabular dysplasia	Assessment of routine imaging
	Re-intervention rate	Number of infants who require further bracing or surgery once randomized (excluding the weaning intervention)
	Impact on the family unit	EMBRACE comparison between the groups
	Number of hospital appointments	To compare the total number of DDH-related hospital appointments between groups post-randomization
	Adverse advents	Foreseeable adverse events

DDH, developmental dysplasia of the hip; EMBRACE, Evaluation Measure for BRACe Experience; VAS, visual analogue scale; WINDY, Weaning in INfant hip Dysplasia.

### Participant schedule

Parents will complete electronic questionnaires at baseline and at two, four, and six weeks post-randomization. Clinical data, including routine imaging findings, AEs, and re-intervention, will be collected at four and 12 weeks post-randomization. The data collection schedule is summarized in Table II.

**Table II.** Data collection schedule.

	**Timepoint**				
**Outcome measure**	**Baseline**	**Two weeks**	**Four weeks**	**Six weeks**	**12 weeks**
Demographic and health data (clinician reported)	X				
BSCOS core measurement set (clinician reported)	X				
Acetabular dysplasia on routine imaging (clinician reported)					X
EMBRACE (parent reported)	X	X	X	X	
VAS (parent reported)	X	X	X	X	
Self-reported compliance (parent reported)		X	X		
Foreseeable complications (clinician reported)			X		

BSCOS, British Society for Children’s Orthopaedic Surgery; EMBRACE, Evaluation Measure for BRACe Experience; VAS, visual analogue scale.

Due to the pragmatic design, participants will continue to attend routine clinical appointments according to local practice, and no additional visits or imaging are required by the study protocol.

## Data management and analysis

### Data collection

Clinical data and parent-reported questionnaires will be collected directly into a secure web-based study database using REDCap. Parents will receive electronic questionnaire links at scheduled timepoints via email. Telephone completion will be available if required. Routine clinical data, including ultrasound findings and treatment details, will be recorded by site staff using local NHS systems and transferred to the study database.

Subject to approvals, routinely collected data from the Smart4NIPE national screening database will be obtained and compared with trial data to assess accessibility, completeness, and accuracy.

### Participant retention

Automated reminders will be sent for incomplete questionnaires, and site staff or the central team may contact parents to encourage completion. Participants may withdraw from the study at any time without affecting clinical care. Reasons for withdrawal will be recorded where available. Data collected before withdrawal will be retained for analysis unless consent is withdrawn. Participants will be considered lost to follow-up if questionnaires are not completed and contact cannot be re-established despite reasonable attempts.

### Statistical analysis

The study will be analyzed and reported in accordance with CONSORT guidance for pilot and feasibility trials.^19^ Analyses will be primarily descriptive and will focus on estimating feasibility parameters rather than hypothesis testing. Recruitment rates, proportions eligible and randomized, retention, compliance, and data completeness will be reported with confidence intervals where appropriate.

Baseline characteristics will be summarized descriptively by treatment arm. EMBRACE measurement properties will be evaluated using standard psychometric methods, including floor and ceiling effects, internal consistency, and test–retest reliability. Correlation analyses will assess construct validity. Exploratory clinical outcomes, including acetabular development, re-intervention rates, AEs, and healthcare use, will be summarized descriptively by group. All analyses will follow an intention-to-treat principle based on allocated group.

## Monitoring

### Data monitoring

Oversight will be provided by a Trial Management Group (TMG) responsible for day-to-day conduct and a Trial Steering Committee providing independent oversight. Given the low-risk nature of the interventions and short follow-up, a separate Data Monitoring Committee is not planned.

Remote monitoring will review recruitment, data completeness, and data queries. Site monitoring procedures will be implemented in accordance with sponsor requirements.

### Adverse events

AEs are defined as any untoward medical occurrence in a participant during the study period. Expected events include skin irritation, pressure areas, avascular necrosis, femoral nerve palsy, inferior dislocation, brachial plexus injury, and pseudo-paralysis. Serious AEs (SAEs) will be reported in accordance with regulatory requirements to the sponsor and ethics committee where applicable. All AEs will be summarized descriptively by treatment arm.

## Discussion

There is currently no high-quality evidence to guide how Pavlik harness treatment should be discontinued in infants with DDH and clear variation in clinical practice.^8,9^ Weaning is widely used but poorly defined, with heterogeneity in regimens. This study evaluates the feasibility of a standardized weaning intervention developed through previous work with clinicians and families.

A key objective is to determine whether families and clinicians are willing to support randomization between weaning and immediate cessation, and whether the intervention is deliverable and adhered to in routine practice. These findings will be critical in informing the design of a definitive trial.

The study also evaluates important methodological components for future DDH trials. This includes assessing the feasibility of using routinely collected Smart4NIPE data for outcome collection, which may reduce research burden and improve trial efficiency. In addition, the EMBRACE measure will be further evaluated to determine its suitability as a parent-reported outcome in this setting.

As a feasibility study, WINDY is not powered to assess clinical effectiveness. However, exploratory clinical outcomes will inform outcome selection for a future definitive trial, where longer-term radiological measures are likely to be primary endpoints.

More broadly, there is a recognized need for high-quality trials in DDH, particularly in relation to brace treatment. This study will inform the design of a definitive multicentre trial of brace weaning and provide methodological insights relevant to future studies, including the use of parent-reported outcomes and routinely collected data.

### Ethics and dissemination

The study will be conducted in accordance with Good Clinical Practice and the Declaration of Helsinki.^20^ Ethical approval and Health Research Authority approval have been obtained before study commencement (Integrated Research Application System no. 354869). Local confirmation of capacity and capability will be obtained at each participating site before recruitment begins.

### Funding and sponsorship

The study is funded through a National Institute for Health and Care Research Doctoral Research Fellowship (no. 303304). The University of Liverpool is the study sponsor.

### Protocol amendments

This manuscript presents an abridged version of the study protocol; the full protocol and all iterative amendments is available on ISRCTN. Any future amendments will be submitted for approval to the sponsor, ethics committee, and regulatory authorities as required before implementation.

### Patient confidentiality

Participants will be assigned a unique study identification number. Identifiable information will be stored securely and separately from research data in accordance with data protection regulations. Access to identifiable data will be restricted to authorized study personnel. Data will be retained and archived in accordance with sponsor policy.

### Patient and public involvement and engagement

Patient and public involvement has informed the study throughout its development. Parents contributed to study design, intervention selection, and outcome measure development, including EMBRACE. A parent representative is a member of the TMG and will contribute to study oversight and dissemination activities. Parent representatives have been consulted regarding study materials and will be involved in the development of dissemination outputs to ensure accessibility and relevance to families.

### Dissemination

Study findings will be disseminated through peer-reviewed publications and presentations at national and international conferences. A plain-language summary will be provided to participating families and shared through relevant patient organizations and professional networks. The protocol and results will be published to maximize transparency and support the design of a future definitive trial.

**Take home message**

- In infants with developmental dysplasia of the hip, Pavlik harness treatment is widely used, but there is variation in how the brace is discontinued and no randomized evidence to guide practice.

- We need to know whether gradual weaning offers any benefit compared with immediate cessation, and how different approaches affect families.

- This feasibility study will inform the design of a definitive multicentre trial addressing an important research priority identified by clinicians and families.

## Data Availability

The data that support the findings for this study are available to other researchers from the corresponding author upon reasonable request.
